# Regulation of human and mouse bystander T cell activation responses by PD-1

**DOI:** 10.1172/jci.insight.173287

**Published:** 2023-09-22

**Authors:** Catherine T. Le, Logan V. Vick, Craig Collins, Cordelia Dunai, Michael K. Sheng, Lam T. Khuat, Isabel Barao, Sean J. Judge, Ethan G. Aguilar, Brendan Curti, Maneesh Dave, Dan L. Longo, Bruce R. Blazar, Robert J. Canter, Arta M. Monjazeb, William J. Murphy

**Affiliations:** 1 Department of Dermatology, School of Medicine, and; 2 Department of Surgery, University of California, Davis, Sacramento, California, USA.; 3 Masonic Cancer Center, and Division of Blood and Marrow Transplantation, Department of Pediatrics, University of Minnesota, Minneapolis, Minnesota, USA.; 4 Earle A. Chiles Research Institute at the Robert W. Franz Cancer Center, Portland, Oregon, USA.; 5 Department of Internal Medicine, Division of Gastroenterology, School of Medicine, University of California, Davis, Sacramento, California, USA.; 6 Department of Medicine, Division of Hematology, Brigham and Women’s Hospital, Harvard Medical School, Boston, Massachusetts, USA.; 7 Department of Radiation-Oncology and; 8 Department of Internal Medicine, Division of Hematology and Oncology, University of California, Davis School of Medicine, Sacramento, California, USA.

**Keywords:** Immunology, Adaptive immunity, Cytokines, T cells

## Abstract

Bystander activation of memory T cells occurs via cytokine signaling alone in the absence of T cell receptor (TCR) signaling and provides a means of amplifying T cell effector responses in an antigen-nonspecific manner. While the role of Programmed Cell Death Protein 1 (PD-1) on antigen-specific T cell responses is extensively characterized, its role in bystander T cell responses is less clear. We examined the role of the PD-1 pathway during human and mouse non–antigen-specific memory T cell bystander activation and observed that PD-1^+^ T cells demonstrated less activation and proliferation than activated PD-1^–^ populations in vitro. Higher activation and proliferative responses were also observed in the PD-1^–^ memory population in both mice and patients with cancer receiving high-dose IL-2, mirroring the in vitro phenotypes. This inhibitory effect of PD-1 could be reversed by PD-1 blockade in vivo or observed using memory T cells from PD-1^–/–^ mice. Interestingly, increased activation through abrogation of PD-1 signaling in bystander-activated T cells also resulted in increased apoptosis due to activation-induced cell death (AICD) and eventual T cell loss in vivo. These results demonstrate that the PD-1/PD-Ligand 1 (PD-L1) pathway inhibited bystander-activated memory T cell responses but also protected cells from AICD.

## Introduction

T cells are generally activated through recognition of their cognate antigen presented in the context of an MHC alongside costimulatory signaling and cytokine, allowing them to then undergo expansion, mediate effector functions, and then convert to long-lived memory cells. Engagement of both activating and inhibitory signals are required to fine tune and regulate these responses. Long-lived antigen-experienced memory T cells eventually become the predominant lymphocyte population with age due to thymic involution, reducing naive T cell output, natural homeostatic T cell expansion, and exposure to continuous antigenic challenges (1). Due to expression of both CD122 and CD132 receptors involved in cytokine signaling, memory T cells also gain the ability to be activated in the absence of T cell receptor (TCR) triggering and by IL-2 or IL-15 cytokine signaling alone, bypassing the need for TCR engagement in a process called “bystander activation.” This antigen-nonspecific activation can be induced during inflammatory conditions, which is dependent on strong stimuli,such as acute viral infections or cancer immunostimulatory therapies, to maintain function and survival (2–4). This property has been demonstrated and exploited clinically with the generation and use of lymphokine-activated killer (LAK) or cytokine-induced killer (CIK) cells, in which both activated T and NK cells mediate antitumor effects, in part mediated by NKG2D ligand recognition (5). The presence and function of bystander-activated tissue-resident memory T cells is being more widely recognized in multiple human disease states, such as viral infection (6) and cancer (7), as a means for bridging and amplifying innate and adaptive effector functions (4, 8). The roles of bystander T cells may become more predominant during aging, at a time when the TCR repertoire may reflect decreasing diversity and increased prevalence of memory T cells due to loss of naive T cells (9). However, how the memory T cell pool is able to later mount antigen-specific responses via bystander activation despite contraction of the stimuli remains unclear (10–13). This same high-cytokine milieu, such as that observed in sepsis or cytokine storm, can also concurrently promote apoptosis of T cells by activation-induced cell death (AICD), leading to cell loss (14). The consequences of repeated bystander activation on AICD and memory T cell numbers may be more pertinent in aging where a finite T cell pool exists.

Immune checkpoint inhibition (ICI) of the Programmed Cell Death Protein 1 (PD-1)/PD-Ligand 1 (PD-L1) axis has achieved considerable success in treating an ever-increasing variety of cancer types and is being increasingly applied both as a single modality and in combination therapies (15). PD-1 is immediately expressed upon TCR engagement during priming but is also found on chronically stimulated memory T cells where it impairs cytokine production, lytic potential, and proliferative ability, in a state called “exhaustion” (16, 17). Direct blockade of this pathway targeting either PD-1 or PD-L1 has been demonstrated to markedly augment antigen-specific T cell effector functions and increase antitumor efficacy. Given the therapeutic efficacy and acceptable off-target toxicities in many patients, administration of checkpoint blockade targeting the PD-1/PD-L1 pathway has been extended in duration in some cancers for years (18). PD-1 is expressed on various percentages of normal human memory T cells and increases with age and chronic exposure to pathogens. However, the role of PD-1/PD-L1 in regulating bystander T cell activation and on the overall memory T cell pool has not been studied.

We hypothesized that the PD-1/PD-L1 axis, acting in its normal physiological role to dampen activation and prevent autoimmunity, also suppresses the function of the population of memory cells expressing PD-1 during bystander activation, potentially dampening their effector responses. We demonstrate here that both mouse and human memory T cells exhibit similar effects after bystander activation in both in vitro and in vivo model systems and that PD-1^+^ T cells are indeed inhibited compared with PD-1^–^ memory T cells. Furthermore, blockade of the PD-1/PD-L1 pathway could restore bystander T activation responses in the PD-1^+^ populations. However, this also was correlated with increased T cell loss due to AICD. These results suggest that the combination of PD-1 inhibition and bystander T cell activation, while increasing T effector responses, may ultimately impair the ability of the host to sustain responses or maintain memory T cell populations.

## Results

### Human and mouse bystander-activated memory T cells have a distinct phenotype and differential effects on activation are observed on PD-1^+^ versus PD-1^–^ memory T cell subsets.

Memory, but not naive, T cells can respond directly to cytokine signaling alone due to their expression of both CD122 and CD132 receptors in a process called bystander activation. The stimulation of this receptor complex bypassing TCR signaling results in antigen-nonspecific activation not resulting in the induction of CD25 and PD-1 and effector functions via NKG2D triggering. Systemic immunostimulatory therapies, such as high-dose (HD) IL-2 administration in both mice and humans as well as acute inflammation that occurs after acute viral infections, can induce robust bystander T cell activation, overcoming a lack of CD25 expression on memory T cells not undergoing TCR signaling yet allowing for effector functions such as NKG2D-mediated lysis of tumor or virally infected cells. This may play an important role in amplifying T cell responses as well as being a potential bridge between innate and adoptive immunity (4, 15, 19).

**Figure 1:**
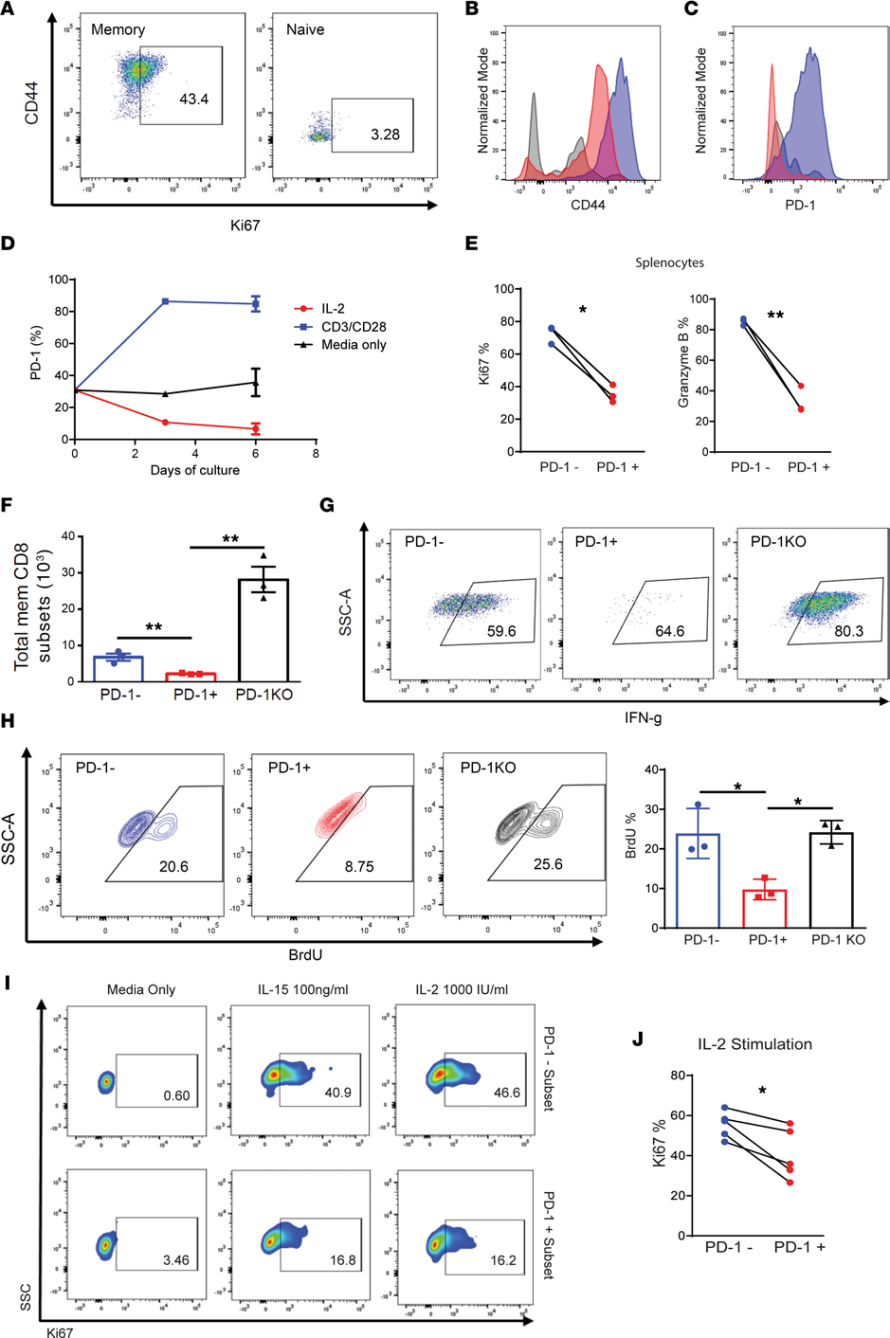
Human and mouse bystander-activated memory T cells have a distinct phenotype following activation and differential effects are observed on PD-1^+^ versus PD-1^–^ memory subsets in vitro. (**A**) Flow Cytometry of Ki67 expression for IL-2 (1,000 IU) stimulated memory and naive murine T cells. (**B** and **C**) Flow cytometry MFI histograms of in vitro cultures of mouse T cells stimulated with IL-2, CD3/CD28, or media alone. (**D**) In vitro time course of PD-1 expression over time across stimulations with IL-2, CD3/CD28, or media alone (*n* = 3). (**E**) Ki67 and Granzyme B expression from PD-1^+^ and PD-1^–^ T cells cultured from murine splenocytes stimulated with IL-2 in vitro (*n* = 3). (**F**) Total number of memory CD8 T cells following IL-2 in vitro stimulation (*n* = 3). (**G**) Flow Cytometry plots of IFN-γ staining in PD-1^–^ and PD-1^+^ WT T cells and PD-1^–/–^ T cells. (**H**) Representative flow cytometry staining of BrdU incorporation within PD-1^–^ and PD-1^+^ WT T cells and PD-1^–/–^ T cells and quantified results (*n* = 3). (**I**) Representative Ki67 staining of PD-1^–^ and PD-1^+^ human T cells stimulated with IL-2, IL-15, or maintained in media alone. (**J**) Quantified flow cytometry Ki67 percentages of PD-1^–^ and PD-1^+^ human T cells stimulated with IL-2 (*n* = 4). All experiments depicted are representative of at least 2 experiments. Two-tailed paired Student’s *t* test (**E** and **J**) used to compare 2 groups. One-way ANOVA with Tukey’s post hoc test for comparison of 3 or more groups (**F** and **H**). *P < 0.05, **P < 0.01.

The differential markers of bystander activation (using IL-2 alone) versus TCR-triggered signaling (with anti-CD3/28 or use of mitogen) was first assessed with both mouse and human T cells. In vitro culture of mouse splenocytes with IL-2 robustly activated and expanded the memory (CD44^+^/CD62L^–/+^) T cell and not naive T cell subsets (Figure 1A). In agreement with previous literature, the amounts of cytokine, such as IL-2, needed to sufficiently induce bystander activation and proliferation ex vivo are of orders of magnitude higher compared with antigen-specific T cell proliferation where induction of CD25 results in the high-affinity IL-2 receptor complex (Supplemental Figure 1A; supplemental material available online with this article; https://doi.org/10.1172/jci.insight.173287DS1) (20). In marked contrast to anti-CD3/CD28 stimulation, in which all the T cells now expressed PD-1 and CD25, IL-2 culture alone did not increase PD-1 expression in the activated memory T cells, with similar levels being present after activation (Figure 1, B and C). Interestingly, when assessed for PD-1 expression over time, there was a decrease in the PD-1^+^ population with IL-2 expansion after 6 days due to increased PD-1^–^ T cell expansion (Figure 1D). This was correlated with lower proliferative markers (ki67) and activation markers (granzyme B) in the PD-1^+^ bystander-activated memory T cells (Figure 1E and Supplemental Figure 1, B and C). This was also reflected with greater total accumulation of the PD-1^–^ subsets. We then assessed the bystander-activation potential of T cells from PD-1 KO mice and observed a markedly heightened ability of the memory T cells from these mice to respond to IL-2 stimulation/expansion as evidenced by increased total numbers (Figure 1F), increased IFN-γ production (Figure 1G), and proliferative capacity by BrdU uptake (Figure 1H), mirroring the results with the PD-1^–^ T cells in the WT mice. These results indicate that bystander activation of mouse memory T cells using cytokine alone results in differential effects on activation and proliferation with the PD-1^–^ memory T cells exhibiting markedly greater activation. These effects occurred in both CD8^+^ and CD4^+^ memory T cells.

We then assessed the role of PD-1 on bystander activation of human memory T cells. As observed with the mouse T cells, bystander-activated human PD-1^–^ memory CD8 T cells proliferated to a much greater extent than the PD-1^+^ population following IL-2 or IL-15 coculture (Figure 1, I and J). These results then demonstrate that PD-1 inhibits antigen-nonspecific bystander memory T cell-activation responses in both human and mouse memory T cells in vitro.

### Transcriptomic profiling of PD-1^–^ and PD-1^+^ human and mouse memory T cell subsets to bystander activation reveal transcriptomic differences commiserate with the functional effects.

**Figure 2:**
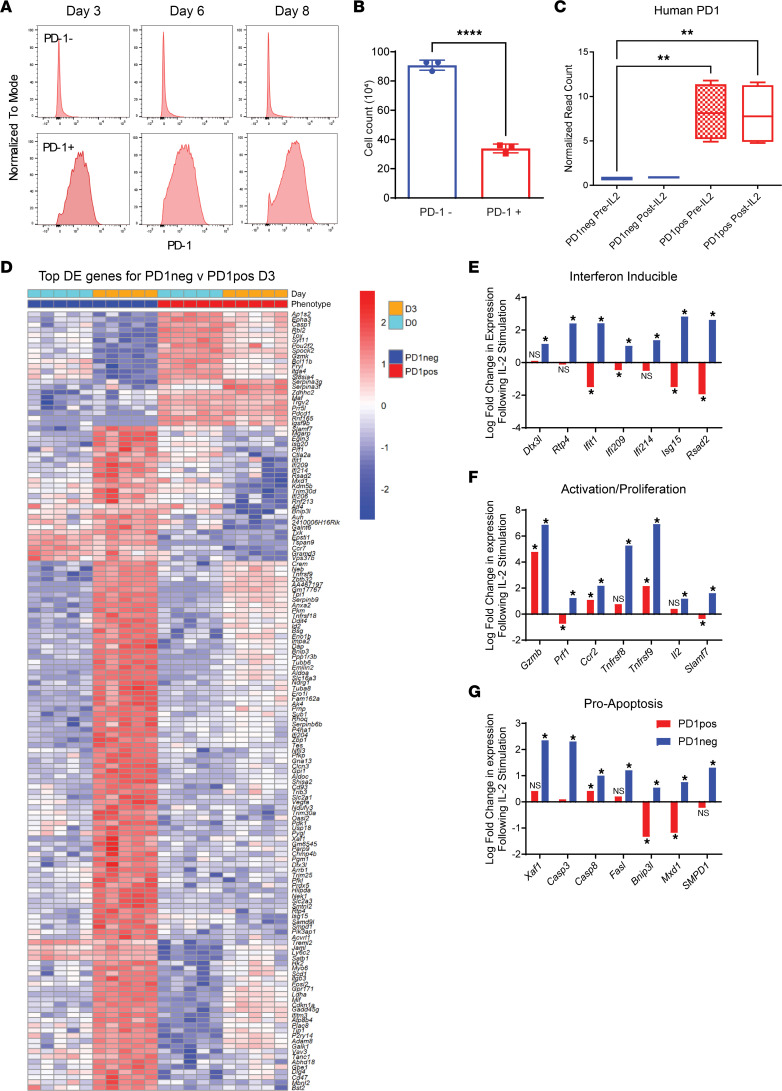
Transcriptomic profiling of PD-1^–^ and PD-1^+^ human and mouse memory T cell subsets to bystander activation. (**A**) Human **PD-1^–^** and PD-1^+^ memory CD8 T cells were sorted and cultured with IL-2 and examined for PD-1 expression by flow cytometry. (**B**) Hemocytometer count of sorted **PD-1^–^** and PD-1^+^ memory T cells cultured with IL-2 on day 11. (**C**) Human *PDCD1* gene normalized read counts pre– and post–IL-2 stimulation. (**D**) Top differentially expressed genes of **PD-1^–^** and PD-1^+^ memory CD8 T cells sorted from mouse splenocytes and cultured for 3 days with IL-2. (**E**) IFN-inducible genes, (**F**) activation/proliferation genes, and (**G**) proapoptosis-associated genes log fold change in expression post–IL-2 stimulation from transcriptome analysis. Data depicted are representative of at least 2 experiments. Sample size *n* = 4 for human donors in **A**–**C** and *n* = 5 for individual mice in **D**–**G**. *n* = 3 for technical replicates in **B**. A 2-tailed paired Student’s *t* test was used for comparison of 2 groups in **B**. An ordinary 1-way ANOVA with Tukey’s multiple comparisons test was used for comparison of multiple groups in **C**. *P < 0.05, **P < 0.01, ****P < 0.0001.

We next examined the molecular pathways of purified human PD-1^+^ and PD-1^–^ memory CD8 T cell subsets after in vitro bystander activation with IL-2. Interestingly, after isolation and 8 days of IL-2 in vitro culture, both subsets maintained their initial baseline PD-1^+^ or PD-1^–^ expression phenotype (Figure 2A). This addresses an important issue that the PD-1^+^ and PD-1^–^ memory T cells were not undergoing conversion after bystander activation and expansion. Consistent with the in vitro results with mixed populations, both greater proliferation and total cell numbers of PD-1^–^ T cells were observed compared with PD-1^+^ T cells after in vitro culture with IL-2 (Figure 2B). Transcriptome analysis further established the stability of PD-1 expression with IL-2 activation within the subsets, as the PD-1 negative population remained negative (Figure 2C). Analysis of human PD-1^–^ and PD-1^+^ T cells also revealed that the PD-1^–^ T cells had increased Ki67 expression compared with PD-1^+^ T cells, and, notably, the PD-1^–^ T cells also had significant differences in expression of CD95 following IL-2 stimulation, suggesting a potential link to AICD (Supplemental Figure 2, A–C). We then assessed patterns in mouse bystander-activated T cells under similar conditions. Sorted PD-1^+^ and PD-1^–^ mouse memory CD8 T cells after culture and expansion also demonstrated different and similar gene expression profiles with several genes being differentially upregulated following IL-2 stimulation comparable to the human data (Figure 2D and Supplemental Figure 2, D and E). IL-2 stimulation caused marked increases in the PD-1^–^ subsets compared with the PD-1^+^ populations with IFN-inducible gene families as well as activation/proliferation and even proapoptosis gene families illustrated through fold change in gene expression following IL-2 stimulation (Figure 2, E–G). These results demonstrate that, in agreement with the phenotypic and functional differences, PD-1 remains consistent within PD-1^–^ and PD-1^+^ subsets and exerts transcriptional profiling effects on human and mouse bystander-activated memory T cells as evidenced by transcriptomic analyses showing significant activation, cytokine, cell proliferation, and apoptotic pathway gene differences based on PD-1 expression on purified subsets.

### Differential activation responses in PD-1^+^ and PD-1^–^ bystander-activated T cells in mice and patients undergoing HD IL-2 treatment.

**Figure 3:**
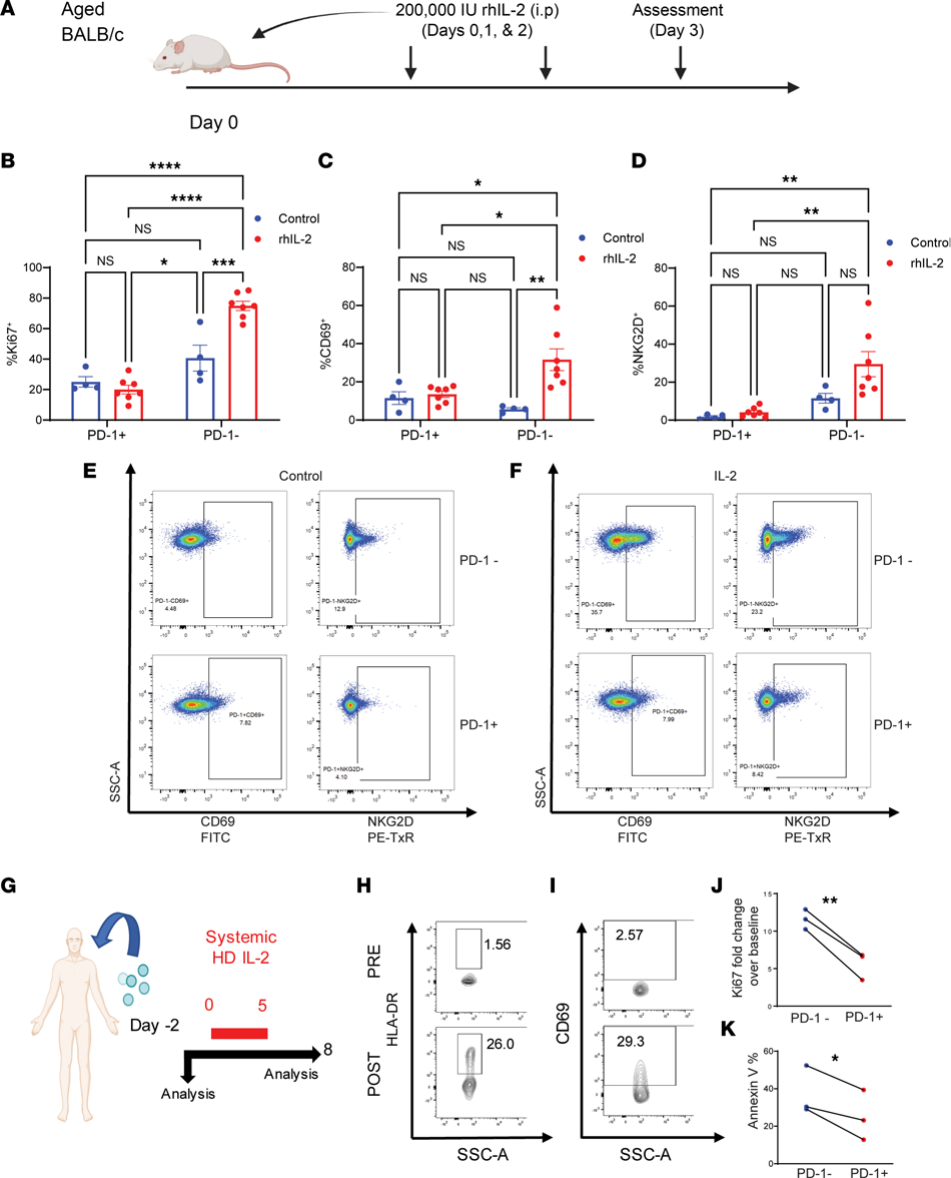
Differential activation responses in PD-1^+^ and PD-1^–^ bystander-activated T cells in mice and patients undergoing HD IL-2 treatment. (**A**) Experimental Schema: aged mice were injected with recombinant human IL-2 i.p. daily and assessed for bystander activation (*n* = 4–7). (**B**) Ki67 percentage of CD3 T cell subsets. (**C**) CD69 percentages of CD3 T cells. (**D**) NKG2D of CD3 T cells. (**E** and **F**) Corresponding representative flow cytometry plots of CD69 and NKG2D staining of PD-1–negative and positive populations in IL-2– or control-treated groups. (**G**) HD IL-2 regimen: Patients were treated with 600,000 IU per kilogram IV every 8 hours for 14 planned doses starting on day 0. PBMCs were collected on day –2 and day 8 and analyzed. (**H**) HLA-DR percentage of T cells before and after treatment within 1 individual patient. (**I**) CD69 percentage of T cells before and after treatment. (**J**) Differential Ki67 upregulation in PD-1**^–^** and PD-1^+^ subsets following treatment over baseline. (**K**) Quantified annexin V percentages of live PD-1**^–^** and PD-1^+^ subsets following treatment. One-way ANOVA with Tukey’s multiple comparison test was used to compare multiple groups in **B**–**D**. Two-tailed paired Student’s *t* tests were used to compare 2 groups in **J** and **K**. *P < 0.05, **P < 0.01, ***P < 0.001, ****P < 0.0001.

We then wanted to ascertain the role of PD-1 on human bystander T cell activation responses in vivo in response to the same cytokine alone stimuli. This was assessed by examining the responses of PD-1^+^ and PD-1^–^ memory CD8 T cell populations in mice receiving HD IL-2 (200,000 IU daily for 3 days (Figure 3A), which has been demonstrated to induce marked T cell activation and expansion, which, in the absence of antigenic challenge, is due to bystander expansion of the memory T cell pool. The results demonstrated, as observed with the in vitro assays, greater activation and expansion of the PD1^–^ memory T cell population compared with the PD-1^+^ T cells based on Ki67, CD69, and NKG2D expression following treatment (Figure 3, B–F).

We then wanted to determine if similar results could be observed with human T cells in vivo. HD IL-2 is currently approved and applied in renal cancer. We obtained PBMC from patients before and after receiving HD IL-2 for cancer treatment (Figure 3G) (21, 22). HD IL-2 clinically has been well demonstrated to induce robust peripheral T cell activation responses and, importantly, mirrors the in vitro T cell profile we have observed regarding the bystander-activated phenotype (CD25^–^, PD-1^–^, and CD69^+^ along with increased effector functions and proliferative capability) (23). This was confirmed and shown by the robust activation of T cells based on HLA-DR and CD69 expression (Figure 3, H and I) compared with baseline expression from patients before receiving treatment. In agreement with the murine in vivo data and human T cell in vitro data, assessment of T cell activation parameters in the patients indicated that PD-1^–^ memory CD8 T cells (CD45RO^+^CD45RA^–^CD8^+^CD3^+^CD56^–^ live cells) from patients receiving HD IL-2 alone exhibited significantly higher fold proliferative indexes than the PD-1^+^ subset (Figure 3J and Supplemental Figure 3A). The activated PD-1^–^ memory CD8 T cells from the patients also exhibited higher apoptosis based on annexin V staining compared with the PD-1^+^ subsets (Figure 3K and Supplemental Figure 3B) following HD IL-2 treatment. These data indicate that similar bystander T cell activation and apoptosis responses in PD-1^+^ and PD-1^–^ T cell subsets occur in both human and mouse systems in vivo following HD IL-2 treatment, mirroring the in vitro data with the PD-1^–^ population showing increased proliferation and activation but also increased AICD compared with the PD-1^+^ population.

### PD-1 downregulates bystander memory T cell responses in vivo during acute systemic viral infection in mice and its reversal using checkpoint blockade.

**Figure 4:**
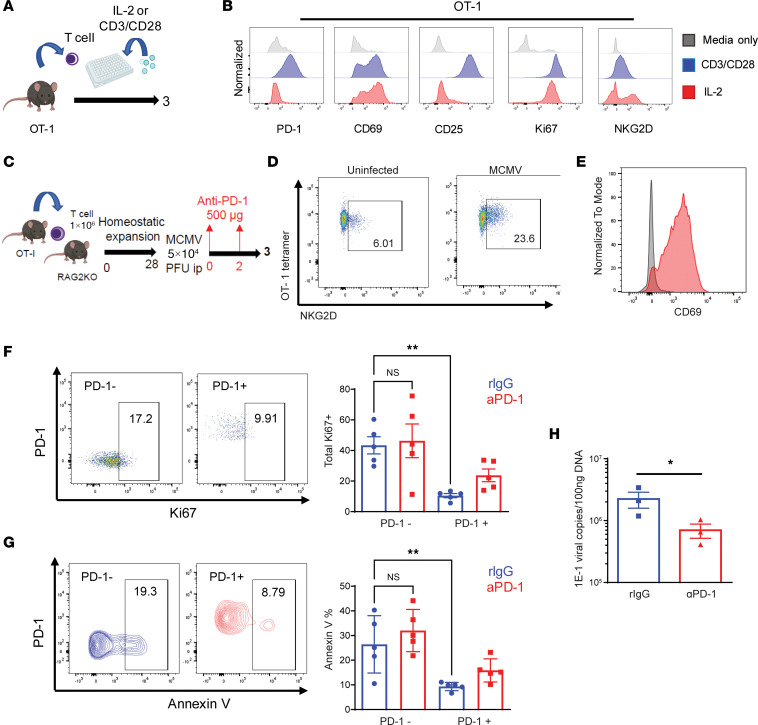
PD-1 downregulates bystander memory T cell responses in vivo during acute systemic viral infection in mice and its reversal using checkpoint blockade. (**A**) Experimental Schema: T cells were isolated from OT-1 spleen and cultured in vitro with IL-2 or CD3/CD28 for 3 days. (**B**) Histogram PD-1, CD69, CD25, Ki67, and NKG2D with culture. (**C**) Experimental schema depicting adoptive transfer of OT-1 Memory CD8 T cells into RAG2^–/–^ mice followed by challenge with MCMV and anti–PD-1 or IgG treatment. (**D**) Representative flow plot of NKG2D percentage of OT-1 T cells in spleen on day 3. (**E**) Histogram plot of CD69 percentage of OT-1 T cells in spleen on day 3. (**F**) Representative flow plot of Ki67 percentage of PD-1**^–^** and PD-1^+^ subsets in liver on day 3 and corresponding quantification. (**G**) Representative flow plots of annexin V percentage of PD-1**^–^** and PD-1^+^ subsets in liver on day 3 and corresponding quantification. (**H**) MCMV viral copies in liver in mice receiving IgG or anti–PD-1 treatments. Experiments are representative of at least 2 experiments and sample size of individual mice depicted is *n* = 5 in **F** and **G** and *n* = 3 in **H**. One-way ANOVA with Tukey’s multiple comparison test was used for comparison of multiple groups in **F** and **G**. Two-tailed unpaired Student’s *t* tests were used to compare 2 groups in **H**. *P < 0.05, **P < 0.01.

The impact of PD-1 on bystander-activated T cells was next assessed in different in vivo models. To definitively demonstrate bystander-activation effects attributed to non–antigen-specific T cell activation, memory CD8^+^ T cells from TCR-transgenic OVA-specific OT-1 (C57BL/6-Tg[TcraTcrb]1100Mjb/J) mice were used for assessment in vivo using a viral challenge model. The phenotype of memory OT-1 T cells following IL-2 culture in vitro demonstrated an identical phenotype (no induction of either PD-1 or CD25) compared with anti-CD3/CD28 stimulation (Figure 4, A and B). Purified CD8^+^ OT-1 T cells were sorted and then adoptively transferred into immune-deficient RAG2 KO (B6[Cg]-Rag2^tm1.1Cgn^/J) mice, which were then infected with murine cytomegalovirus (MCMV) (Figure 4C). This acute infection results in high inflammatory cytokine responses and allows for elevated memory bystander T cell activation. The transferred OT-1 memory T cells were bystander activated as shown by increased CD69 and NKG2D expression (Figure 4, D and E) (19, 24). Analogous to the in vitro studies, the PD-1^–^ subset of the transferred OT-1 CD8^+^ T cells proliferated to a significantly greater extent than the PD-1^+^ population within the liver (Figure 4F). Also mirroring the previous studies with human T cells, along with increased activation, the PD-1^–^ OT1 cells also had greater apoptosis as determined by annexin V staining (Figure 4G). We then assessed the blocking of PD-1 in vivo on the bystander-activated T cell responses in these cells. Concurrent viral infection and anti–PD-1 treatment resulted in a marked increase in proliferative responses of PD-1^+^ OT-1 T cells but had no effect on proliferative responses in the PD-1^–^ population, demonstrating the specific effects of PD-1 blockade and the ability to override the inhibitory effects (Figure 4F). Importantly, the administration of anti–PD-1 in mice receiving the OT-1 T cells also resulted in lower viral titers, indicating that increased bystander-mediated effector functions were also occurring (Figure 4H). Similar to the in vitro activation data using PD-1^–/–^ T cells, concurrent anti–PD-1 treatment also resulted in an increased apoptosis in the PD-1^+^ T cells by AICD, and this was not observed in the PD-1^–^ OT-1 T cells following infection, also demonstrating the specificity of the blockade on the PD-1^+^ cells with both activation and AICD increases (Figure 4G). Thus, PD-1 inhibits antigen-nonspecific bystander T cell activation responses in vivo following viral infection but also suppresses AICD, and these can be overridden with checkpoint blockade, resulting in increased activation and antiviral effector functions in the complete absence of antigen-specific T cell activity.

### Loss of PD-1 signaling increases early functionality but results in increased AICD with bystander activation following viral challenge.

**Figure 5:**
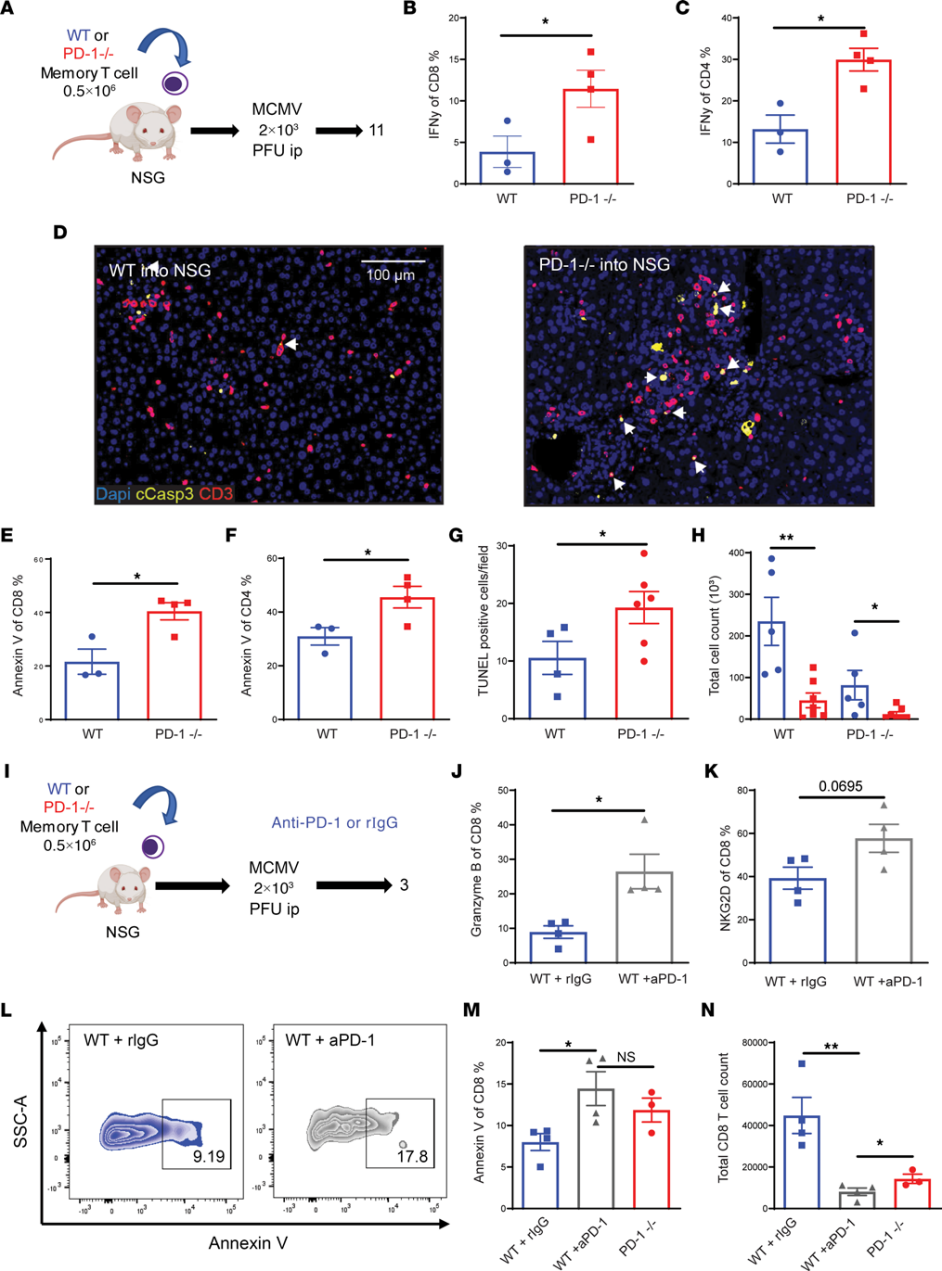
Loss of PD-1 signaling increases early functionality but results in decreased long-term survival with bystander activation. (**A**) Experimental schema: Memory T cells from WT BM chimera or PD-1^–^/^–^ BM chimera were adoptively transferred into NSG mice, which were then infected with MCMV 2 × 10^3^ PFU and assessed on day 11 (*n* = 3–4). (**B**) IFN-γ percentage of CD8 and CD4 T cells in spleen. (**C**) Annexin V percentage of live CD8 and CD4 T cells in the spleen. (**D**) Multiplex immunofluorescence (IF) staining of activated caspase-3 and CD3 in the liver. White arrows indicate activated caspase-3^+^CD3^+^ cells. Scale bar: 100 μm. (**E** and **F**) Annexin V percentages of CD4 and CD8 T cells. (**G**) Quantification of average TUNEL^+^ cells/field in liver. (**H**) Total CD8 and CD4 T cells in spleen on day 11. (**I**) Experimental schema: Memory T cells from WT BM chimera were adoptively transferred into NSG, which were then infected with MCMV 2 × 10^3^ PFU and treated with anti–PD-1 blockade or rat IgG control on days 0 and 2. Analysis was performed on day 3. (**J**) Granzyme B percentage of CD8 T cells. (**K**) NKG2D percentage of CD8 T cells. (**L**) Representative flow plots of annexin V staining of live memory CD8 T cells in the spleen. (**M**) Annexin V percentage of live CD8 T cells in the spleen. (**N**) Total CD8 T cells in spleen on day 3. Data depicted are representative of at least 2 experiments. Bar graphs depict sample size of individual mice. Two-tailed unpaired Student’s *t* test in **B**–**D**, **E**–**H**, **J**, and **K** used to compare 2 groups. One-way ANOVA with Tukey’s post hoc test for comparison of 3 or more groups in **M** and **N**. *P < 0.05, **P < 0.01.

The role of PD-1 on virally induced bystander-activation responses using non-TCR transgenic memory T cell responses was then assessed by adoptively transferring sorted memory (CD44^+^) T cells from WT and PD-1 KO mice into immunodeficient NSG (NOD.Cg-Prkdc^scid^ Il2rg^tm1Wjl^/SzJ) mice. Memory T cells have been previously demonstrated incapable of inducing primary antigen responses, even in allogeneic situations. Prior to sorting, WT and PD-1 KO T cells were evaluated by flow assessing phenotype differences in memory profile and confirming PD-1 expression and then underwent a purity check after sorting to validate cell populations prior to transplant (Supplemental Figure 4, A–F). The NSG mice were then infected with MCMV (Figure 5A). Comparable to the effects using OT-1 T cells, the T cells recovered from PD-1^–^ donors demonstrated increased bystander responsiveness as indicated by increased IFN-γ production with both CD4^+^ and CD8^+^ T cells (Figure 5, B and C). Also, in agreement with the in vitro and human HD IL-2 data, there was also increased apoptosis as determined by caspase immunofluorescence staining, annexin V flow cytometry staining, and TUNEL IHC (Figure 5, D–G and Supplemental Figure 5A). The subsequent loss of T cell numbers by apoptosis was made manifest by greater loss of CD4^+^ and CD8^+^ PD-1 KO T cells over time (Figure 5H).

We then assessed the effects of anti–PD-1 on the ability of the memory T cells to respond following viral infection and bystander activation using the same model (Figure 5I). In accordance with previous OT-1 studies, following infection, anti–PD-1 treatment resulted in the CD8 T cells exhibiting higher early T cell activation indicated by increased granzyme B and NKG2D expression at day 3 (Figure 5, J and K). However, WT T cells from mice receiving anti–PD-1 or receiving PD-1 KO donor T cells had significantly greater apoptosis (Figure 5, L and M), which resulted in significantly greater donor T cell loss after infection (Figure 5N). Subsequently, mice receiving PD-1 KO T cells experienced substantially greater weight loss by week 2 after infection as well as significantly greater viral loads, indicating loss of effector functions (Supplemental Figure 5, B and C). These results demonstrate that absence of PD-1 signaling, whether due to genetic ablation or use of blockade, can initially augment bystander memory T cell activation, proliferation, and function following viral infection due to normalization of the PD-1^+^ subset to that of the PD-1^–^ T cells. However, this increased activation potential using PD-1^–/–^ T cells or with checkpoint blockade also results in greater AICD and eventual T cell loss, which culminates with the reduction of long-term antiviral efficacy.

## Discussion

The clinical application of both HD IL-2 therapy, as well as use in combination with ex vivo generated LAK or CIK cells, has demonstrated antitumor effects for several cancers, albeit with significant toxicities (5, 20). The effector T cells generated in this manner have been demonstrated to mediate non–antigen-specific cytotoxicity in an NKG2D-mediated manner (5, 19, 25). The bystander T cells require the continuous presence of the high cytokine/inflammatory environment to maintain activation and survival, and these cells likely represent a means for early effector responses. The results presented here demonstrate that, similar with effects on antigen-specific T cell responses, the PD-1/PD-L1 axis plays a role in limiting the subset of antigen-nonspecific bystander activation of both human and mouse memory CD4^+^ and CD8^+^ T cells. While both PD-1^+^ and PD-1^–^ memory T cells are activated with cytokine stimulation alone, the proliferative extent of the PD-1^–^ subset was significantly greater in both human and mouse memory cells. Blockade or abrogation of the PD-1 pathway results in increased activation and function of the bystander-activated memory T cells, but also causes increased apoptosis due to AICD leading to overall memory T cell loss. Importantly, this occurred both in vitro and in vivo with clinical results mirroring the preclinical mouse data. Using more stringent models in which no TCR engagement can occur to represent true bystander-activation events, it was revealed that consistency in phenotypes and transcriptomics follow bystander activation in both mouse and human T cells. Notably, while the scope of this investigation was on the role of PD-1 in regulating antigen nonspecific bystander activation, further detailed analysis on PD-1^–^ and PD-1^+^ subsets need to be further explored, particularly in the contexts of different memory subsets, effector signatures, and stemness, especially after blockade, as there may be differential effects contingent on the differentiation stage of the memory population as well as the extent of bystander activation or strength of signal involved.

TCR crosslinking immediately upregulates PD-1 expression, whereas bystander activation only maintains PD-1 expression within the memory T cell subset already expressing it, and this can vary widely on percentages based on numerous factors. The use of TCR transgenic T cells also allowed for definitive assessment of true antigen-nonspecific bystander-activation effects, demonstrating they can mediate protection from viral infections and that purified memory T cells also exhibited comparable behaviors in vitro and in vivo. It is of interest that the human and mouse bystander-activated T cells were remarkably similar in their response to PD-1 signaling, highlighting the evolutionary importance of PD-1 in regulating and maintaining memory T cell populations, especially under bystander-activation conditions where loss of these populations could have significant impact for future antigen-specific responses. This protection from AICD may in part be accomplished by the well-characterized phenomenon of PD-1 in dampening IFN-γ production. IFN-γ–mediated upregulation of CD95 on tumors (26) and virally infected cells (27) has been shown to increase susceptibility to apoptosis, but it is the induction of CD95 on the activated T cells that also limits T cell responses (11). As bystander-activated T cells do not upregulate PD-1 expression, the PD-1^–^–activated bystander T cells more rapidly proliferate and overtake the PD-1^+^ cells both in vitro and in vivo. In this case, the susceptibility of bystander-activated PD-1^–^ T cells to AICD can clear a niche for emerging antigen-specific responses (12) as well as promote T cell contraction after resolution of inflammation or cytokine levels (28). The ability for PD-1/PD-L1 to suppress not only IFN-γ expression but also other effector functions suggests this axis plays a role in maintenance and effector function needs (29). Our data would imply a strong evolutionary role of PD-1/PD-L1 to reduce memory T cell loss due to AICD in the strong inflammatory conditions associated with bystander activation, which is likely important with increasing age and experienced infections due to the predominance of long-lived memory T cell pools. Our studies also highlight the need to use older mice to assess bystander-mediated effects, especially given the low memory T cell compartment with even lower (to negligible) PD-1^+^ cells within that memory pool in younger specific pathogen–free (SPF) mice.

PD-1 and, perhaps other immune checkpoints, such as TIGIT, LAG3, and TIM3, among others, have likely evolved to regulate the changing and dwindling antigen receptor diversity of the T cell compartment with age and continuous antigen stimulation, allowing for maintenance of response capability of a finite pool of memory cells (17, 30, 31). Much of the emphasis on therapeutically targeting PD-1/PD-L1 has centered on the reversal of exhaustion and increasing effector function in cancer with application of such therapeutics being not only increasingly applied but also for longer periods of time. However, the effects of prolonged blockade on overall T cell responses on nominal antigens or with recall responses have not been well characterized in both mouse studies and clinical assessment. Evidence using PD-1^–/–^ mice suggests that impairment of memory T cell responses or maintenance occurs with loss of PD-1 (32, 33), resulting in worsening “exhaustion” and reduced survival months after chronic viral infection (33). The mechanism underlying the impaired function in the mice was unclear but is consistent with our results of increased AICD and T cell loss during states of high stimulation as possibly contributing. In our study, PD-1 deletion or blockade introduced a short-term gain but long-term loss for non–antigen-specific T cell function in the context of strong stimulatory signals, although these studies used adoptive transfer into immunodeficient mice, likely augmenting any effects of this limited pool transferred. The data here pertain to T cells, but this phenomenon may very well apply to other immune cells, such as B cell and myeloid lineage cells, that express PD-1 (34). The role of PD-L1 in regulating apoptosis also has to be further explored, and a recent study implicates PD-L1 in reducing apoptosis of neutrophils in models of sepsis (35). Interestingly, the same strong cytokine signaling responsible for bystander activation of T cells may induce PD-L1 expression on a number of cells, including those of the myeloid lineage or even the tumor itself, providing additional brakes for T cells, yet this would also provide further opportunity for ICI to override inhibitory signaling. However, the impact of PD-L1 in the context of ICI therapy is still contingent upon the nature or types of the immune therapy regimens applied, whether it be antigen specific, bystander activated, or a combination. It is therefore important to understand the potential consequences of removing the immunological “brakes” during events of strong immune stimulation for the immune system’s ability to defend against reinfection or latent infection. It may be possible to determine means of minimizing this loss as well as determining the magnitude of bystander-activation responses needed for sufficient efficacy in various disease states.

The results presented here suggest that long-term application of ICI may have effects on maintenance and function of the finite memory T cell compartment as it also can potentially increase future bystander T cell responses. This may have beneficial, deleterious, or mixed effects in the host. It will be important to gauge the potential impact on critical memory T cells specific to chronic (such as CMV) or acute (such as COVID-19 or influenza) viral infections or vaccines in the aged cancer population undergoing ICI therapy. Tracking these effects may be difficult, as blockade alone does not induce AICD in the absence of strong T cell stimulation. The combinations of ICI with other immunostimulatory regimens may exacerbate this effect. The potential for increased susceptibility from T cell-mediated immunopathology following activation of bystander T cells in patients undergoing long-term ICI should also be considered. Further studies are needed to evaluate the impact of ICI over longer periods of time on overall fitness of memory T cell recall responses in the aged population that rely on these populations.

## Methods

### Mice.

Male and Female C57BL/6NTac and C57BL/6J aged 6–12 weeks old were purchased from Taconic Farms and Jackson Laboratories. OT-1 mice and RAG2 KO mice were purchased from Jackson Laboratories. The NSG mice were purchased from Jackson Laboratories or bred in-house. B6 PD-1^–/–^ (PD-1 KO) mice were provided by Bruce Blazar (University of Minnesota) and originally provided by Tasuku Honjo and Hiroyuki Nishimura (36). In some experiments, B6 PD-1^–/–^ donor mice were used to create BM cell (BMC) chimeras into lethally irradiated congenic recipients and used as a source of T cells 60 days after reconstitution and determination of donor origin (19). All mice were housed in Association for Accreditation of Laboratory Animal Care-approved SPF facilities with free access to food and water and were aged in the facility for use.

### In vitro culture experiments.

Single-cell suspensions were plated in 96-well flat bottom plates at 150,000–200,000 cells per well. Cells were stimulated with recombinant human IL-2 (TECIN Teceleukin, catalog 202-IL), recombinant human IL-15 obtained from the NCI Biological Resources Branch, CD3 (eBioscience, catalog 16-0031-86) for mouse cells, CD28 (eBioscience, catalog 16-0281-86) for mouse cells, or Dynabeads Human T-Activator CD3/CD28 (Gibco, catalog 11161D) for human cells. For BrdU proliferation studies, 10 uM BrdU was add on day 8 of culture and cells were stained for flow cytometry assessment on day 10. Cells were stained according to the manufacturer’s instructions (BD, catalog 559 619) with anti-BrdU FITC provided in kit. Cells were incubated using complete RF10c media containing 10% Nu Serum (Corning, IV Culture, catalog 355504), 2 mM glutamine (Gibco, catalog 25030-081), 1% nonessential aa (Corning, catalog 25-025-CI), 1% penicillin-streptomycin (Corning, catalog 30-002-CI), 5 × 10^–5^ M 2-mercaptoethanol (Sigma-Aldrich, catalog M7522-100), 1 M HEPES buffer (Gibco, catalog 15630-080), and 1 mM sodium pyruvate (Corning, catalog 25-000-CI) at 37°C, 5% CO2.

### Adoptive transfer studies.

OT-1 donors were either immunized with BM-derived DCs pulsed with OVA (OVA 257–264) SIINFEKL peptide (Sigma-Aldrich, catalog 138831-86-4) or used without immunization, as indicated. PD-1^–/–^ BM chimeras and WT BM chimeras were generated by adoptively transferring 25 × 10^6^ BM cells from B6 PD-1^–/–^ or control donor mice into C57BL/6J recipient mice lethally irradiated with a split dose of 650 cGy. PD-1 blockade (29F.1A12; BioXCell, catalog BE0273,) or rat IgG (Jackson ImmunoResearch, catalog 012-000-002) was administered on indicated days with 500 μg on days 0 and 2. Recombinant human IL-2 (TECIN Tecekeyjub) was administered at 200,000 IU per day in NSG mice or 200,000–500,000 IU per day in aged mice as indicated.

### MCMV infection.

MCMV Smith strain was obtained from American Type Culture Collection and maintained by repeated salivary gland passage in BALB/c mice. MCMV was administered i.p. in 0.2 mL of RPMI medium. Quantification of MCMV virus using real-time PCR was performed as previously described (37, 38). Briefly, DNA was extracted from livers using the DNeasy Tissue Kit (Qiagen, catalog 69506) and the MCMV IE1 gene was amplified using the HotStarTaq Master Mix (Qiagen, catalog 203446) and forward and reverse primers. Standard curve was constructed by plotting the Ct value against the log of IE1-containing plasmid, followed by a sum of least square regression analysis. Plasmid was purified and quantified by serial 10-fold dilutions using forward and reverse primers and probe. Target copy numbers in the tissue samples were then calculated using the equation obtained by least square regression analysis. Results were expressed as IE1 gene copies/100 μg of DNA. Data was analyzed on CFX Maestro Software (Bio-Rad).

### Human HD IL-2 regimen.

Blood samples were obtained from patients with metastatic melanoma (NCT 01416831) or renal cell carcinoma (NCT 02306954) enrolled in a randomized Phase II trial and receiving HD IL-2 alone as previously described (21, 22, 25, 26). Briefly, HD IL-2 (Proleukin [aldesleukin], Prometheus Laboratories) was administered at 600,000 IU/kg by i.v. bolus infusion given every 8 hours for 14 planned doses. PBMCs were isolated from blood samples obtained at baseline and day 8 and were cryopreserved for future analysis.

### Isolation cell protocol.

Mouse T cells were isolated using MagniSort Mouse T cell Enrichment Kit (Invitrogen, catalog 8804-6820-74). Mouse T cells were sorted on BD FACSAria II (BD Biosciences). Human T cells were isolated using the MagniSort Human T cell Enrichment Kit (Invitrogen, catalog 8804-6810-74). Human T cells were sorted on the BD Influx Cell Sorter (BD Biosciences).

### Flow cytometry.

Single-cell suspensions were prepared from spleens, livers, lung, or salivary glands. Peripheral blood was collected by tail vein bleeds and lysed with BD Pharm Lyse (BD Bioscience, catalog 555899). Cells were incubated with Fc block (anti-CD16/32 clone 93; BioLegend for mouse; Human TruStain FcX, BioLegend for human). LIVE/DEAD Fixable Aqua Dead Cell Stain Kit (Invitrogen) and Fixable Viability Dye eFluor780 (Invitrogen) were used to stain for dead cells. Apoptosis was assessed by annexin V (PB or PerCp-eFluor710) staining (BioLegend) using Apoptosis Detecting Kit (eBioscience) following the manufacturer’s instructions. For intracellular cytokine staining, cells were incubated at 37° C with 4 L/6 L GolgiStop (BD Biosciences) and 1 L/mL GolgiPlug (BD Biosciences) only or with Phorbol 12-myristate 13-acetate (PMA) and ionomycin for 4 hours before surface staining. Intracellular cytokine staining was performed using Cytofix/Cytoperm kit (BD Biosciences). Flow cytometry data were acquired on an LSR Fortessa flow cytometer (BD Biosciences) and analyzed using FlowJo 10.6.1 software.

For mouse studies, the following fluorochrome-conjugated monoclonal Abs were purchased from BioLegend: APC-anti-CD45 (30-F11), PB or AF700 anti-CD44 (IM7), BV711 or PE anti-CD4 (RM4–5), BV785 or APC anti-CD3 (17A2), BV605 anti-CD8α (53–6.7), PE anti–PD-1 (RMP1-30), AF700 anti-Ki67 (16A8), BV605 or PE anti-CD69 (H1.2F3), PerCP-Cy5.5 anti-CD25 (PC61), FITC or AF647 anti–granzyme B (GB11), and PE-Cy7 anti-CD3 (145-2C11). The following fluorochrome-conjugated monoclonal Abs were purchased from Thermo Fisher Scientific: FITC or APC anti–PD-1 (RMP1–30), PE-Cy7 anti-CD62L (MEL-14), FITC anti-CD8α (KT15), PE-eFluor610 anti-CD314 (NKG2D; CX5), and APC anti-CD44 (IM7). The following were from BD Biosciences: APC-Cy7 anti–IFN-γ (XMG1.2), FITC anti-CD69 (H1.2F3), PE anti-CD8α (53–6.7), and FITC anti-CD95 (Jo2).

For human studies, the following fluorochrome-conjugated monoclonal Abs were purchased from BioLegend: BV785 anti-CD3 (OKT3), FITC anti-CD3 (HIT3a), BV785 or BV605 anti-CD8 (RPA-T8), BV711 anti-CD4 (OKT4), PE anti-CD56 (HCD56), PE/Dazzle anti-CD45RA (HI100), BV421 or APC anti-CD45RO (UCHL1), PE or APC anti–PD-1 (EH12.2H7), APC-Cy7 or BV510 anti–IFN-γ (B27), FITC or PE-Cy7 anti-CD69 (FN50), BV510 anti-CD25 (M-A251), FITC or BV605 anti-CD95 (DX2), PE-Cy7 anti–granzyme B (QA16A02), FITC or AF647 anti–granzyme B (GB11), APC-Cy7 anti-CCR7 (G043H7), and FITC or PerCP eFluor 710 anti–HLA-DR (L243). The following fluorochrome-conjugated monoclonal Abs were purchased from Thermo Fisher Scientific: PerCP-eFluor710 anti-CD69 (FN50), PE anti–PD-1 (MIH4), and APC or FITC or PE-Cy7 anti–Ki-67 (20Raj1). From BD Biosciences, the following was used: AF700 anti-CD25 (M-A251).

### Tetramer staining.

NIH Tetramer Core Facility (Emory University) provided the following tetramers: PE-MHC class I tetramer, consisting of murine H-2Kb complexed to SIINFEKL (OVA257-264) peptide. OVA257-264 tetramer was stained at 4° C for 1 hour prior to cell surface stain. All tetramers were stained at 1:100.

### Multiplex immunostaining and tissue imaging.

FFPE liver 4 μm sections were obtained for immunostaining. To see the expression and activity of caspase-3 in hepatic T cells, we utilized multiplex IHC using a tyramide signal amplification (TSA) method. Sections at 4 μm thickness were cut from FFPE tissues. After deparaffinization and rehydration, initial antigen retrieval was performed in 10 mM citrate buffer using a decloaking chamber (Biocare Medical) for 45 minutes at 125^o^C at 15 psi. Primary Abs were used to active Caspase-3 (R&D System, catalog AF835; 1:200) and CD3 (Cell Signaling, catalog 99940; 1:150). TSA visualization was performed with the Opal 7-color manual IHC kit (NEL811001KT). The cleaved region of caspase-3 was labeled by Opal 570, and anti-CD3 signaling was by Opal 690. Each TSA signal was finished with microwave treatment, and counterstaining was performed by DAPI in Fluoromount-G mounting medium (Thermo Fisher Scientific). Stained slides were scanned using the Vectra 3 Automated Quantitative Pathology Imaging System (Akoya Biosciences). Multiple regions of interest were selected using the Phenochart viewer (Akoya Bioscience) and imaged at x20 objective. To build a spectral library for spectral unmixing, DAPI-only stained sections and single-stained paired slides were prepared. Multispectral images were then unmixed using spectral libraries using the inform Advanced Image Analysis software (Akoya Biosciences).

### TUNEL IHC.

For histological staining, livers were fixed in 10% neutral buffered formalin for 24 hours at room temperature and then placed in 70% ethanol. Tissue was paraffin embedded. Multiple 4 μm sections were cut and stained with H&E stain or the following reagent: rabbit anti–mouse-PD-1 (Clone EPR20665, Abcam). TUNEL assay was completed following manufacturer’s instructions with ApopTag Plus Peroxidase In Situ Apoptosis (Millipore Sigma). Images were captured by Olympus FSX100 all-in-one Fluorescence Microscope (Olympus Life Science).

### RNA-Seq.

Total RNA was extracted from sorted mouse or human T cells using Total RNA Purification Plus Micro Kit (Norgen Biotek) following standard protocols and quantified using Qubit RNA HS kit on a Qubit fluorimeter (LifeTechnologies). RNA integrity was assessed using TapeStation 2200(Agilent). Barcoded 3’ Tag-Seq libraries were created by UCD DNA Technologies Core facility using QuantSeq FWD kit (Lexogen) for multiplexed sequencing according to the recommendations of the manufacturer. The fragment size distribution of the libraries was verified via microcapillary gel electrophoresis on a Bioanalyzer 2100 (Agilent). The libraries were quantified by fluorometry on a Qubit instrument (LifeTechnologies) and pooled in equimolar ratios. The libraries were sequenced on a HiSeq 4000 sequencer (Illumina) with single-end 100 bp reads. Analysis of the sequencing data was performed by UCD Bioinformatics Core. Raw reads were processed with HTStream v.1.1.0 (https://s4hts.github.io/HTStream/); to perform raw sequence data QA/QC, adapter contamination and low-quality bases/sequences were removed. The trimmed reads were aligned to the Mus musculus GRCm38 primary assembly genome with GENCODE v.M23 annotation (for mouse) or to the Homo sapiens GRCh38 primary assembly genome with GENCODE v.32 annotation, using the aligner STAR v. 2.7.0f (39) to generate raw counts per gene. Raw counts per gene were normalized and analyzed for differential gene expression using Bioconductor packages edgeR (39) and limma (40).

### Statistics.

Graphs were made and statistical analyses were performed using GraphPad Prism Version 6.02 (GraphPad Software). Data were expressed as mean SD or SEM, as indicated. One-way or 2-way ANOVA tests were performed with Tukey’s post hoc test or Holm-Sidak’s multiple comparisons testing, as appropriate. The 2-tailed Student’s *t* test was used to compare differences between 2 normally distributed test groups. *P* values of less than 0.05 were considered statistically significant. Statistical outliers were identified using the robust regression and outlier removal (ROUT) test.

### Study approval.

All experimental protocols involving animals were approved by the IACUC of UCD. Signed and informed consent was obtained before enrollment and collection of patient samples and the Providence Health System Regional Institutional Review Board, Oregon, approved the study.

### Data availability.

Original data values can be found in the Supporting Data Values file. Data from RNA-Seq can be found on the NCBI GEO database (accession GSE199615).

## Author contributions

Contribution: CTL and WJM directed the project. CTL, CD, LTK, LVV, AMM, and WJM designed and analyzed the research. CTL, CD, LTK, LVV, IB, SJJ, CC, MKS, and AMM performed experiments and analyzed the results. CTL, LVV, AMM, and WJM cowrote the manuscript. EGA, BC, RJC, MD, DLL, and BRB provided essential reagents and/or design contributions. All coauthors edited the manuscript.

## Supplementary Material

Supplemental data

Supporting data values

## Version 1 09/22/2023

Electronic publication
