# Supplementary material for: Regulation of human and mouse bystander T cell activation responses by PD-1
Source: JCI Insight. 2023 Sep 22;8(18):e173287. doi: 10.1172/jci.insight.173287 (PMC10561715; doi:10.1172/jci.insight.173287)
Supplement: Supplemental data [file jciinsight-8-173287-s193.pdf]

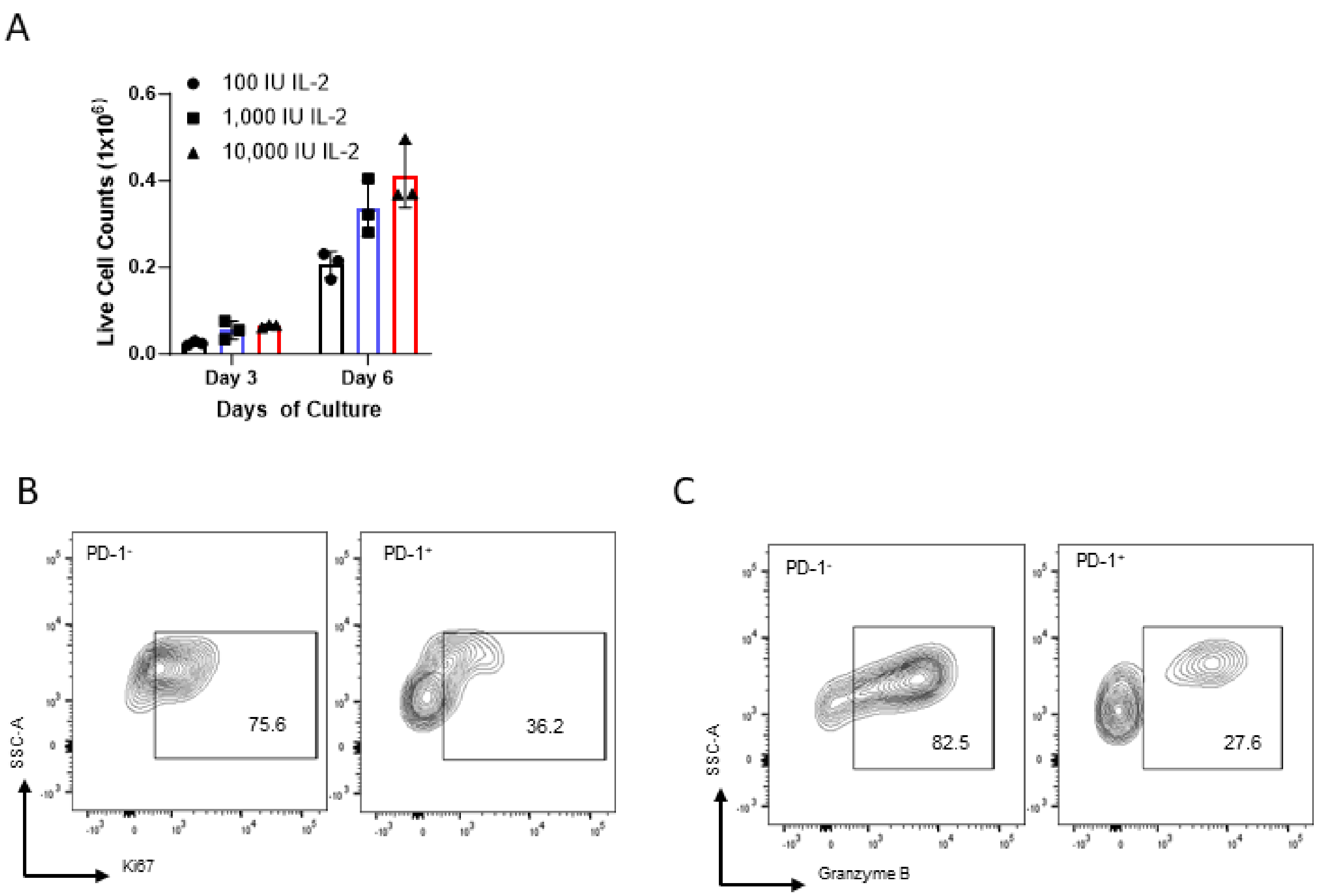

Supplemental Figure 1. A) Live cell counts following in vitro culture of IL-2 at three doses (100 IU, 1,000 IU, and 10,000 IU) at 3 and 6 days. B & C) Flow cytometry plots of Ki67 and Granzyme B expression from PD-1+ and PD-1- T cells cultured from murine splenocytes stimulated with IL-2 in vitro. All experiments depicted are representative of at least two experiments.

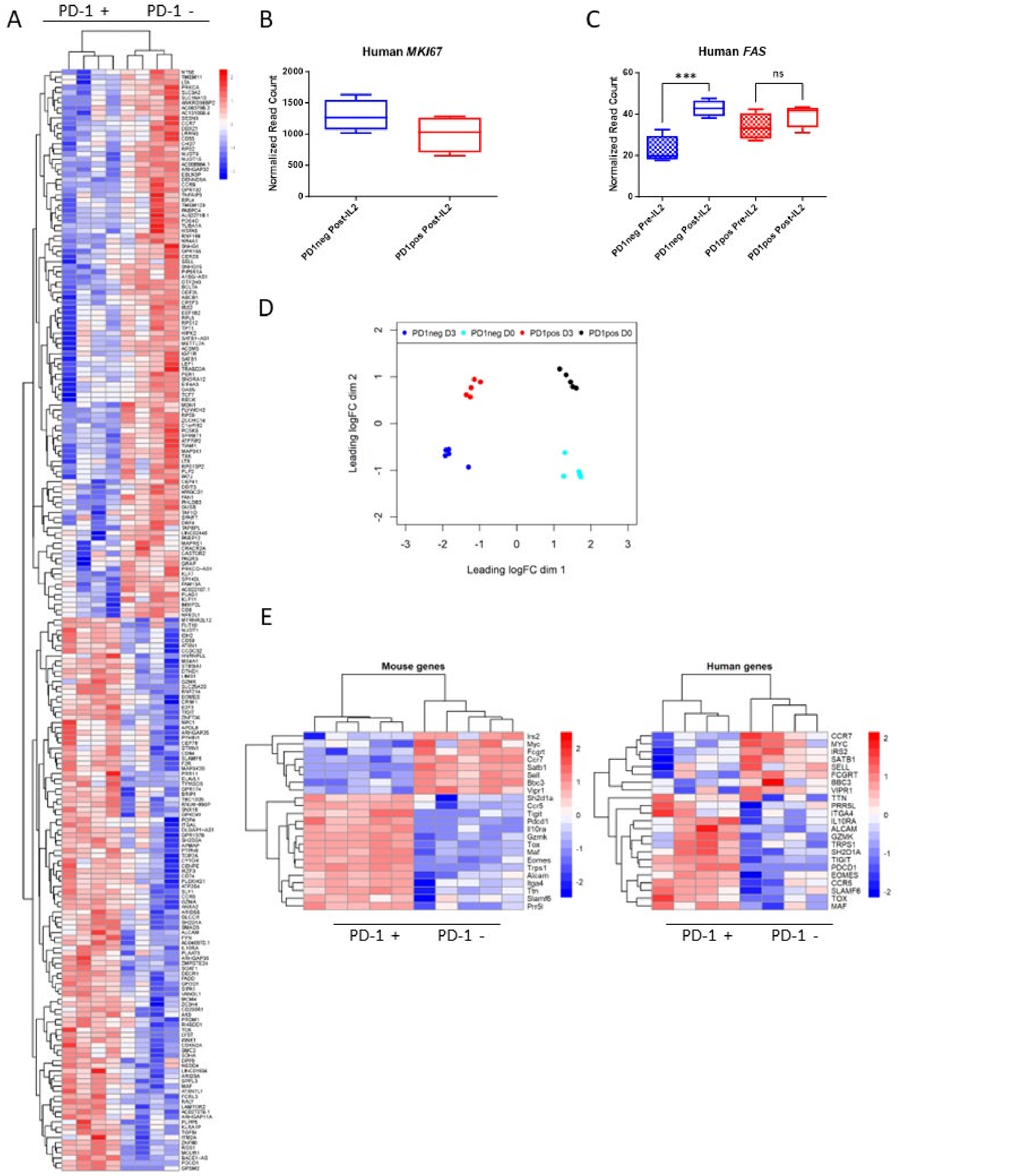

Supplemental Figure 2: A) Top differentially expressed genes form human PD-1- and PD-1+ memory CD8 T. B) Human *MKI67* gene normalized read counts post-IL-2 stimulation. C) Human *FAS* gene normalized read counts pre- and post-IL-2 stimulation. D) PCA plot of sorted mouse PD-1- and PD-1+ CD8 T cells pre and post IL-2 stimulation. E) Comparison of similarly differentially expressed genes between mouse and human PD-1- and PD-1+ memory CD8 T cells. Sample size n = 4 for human donors and n = 5 individual

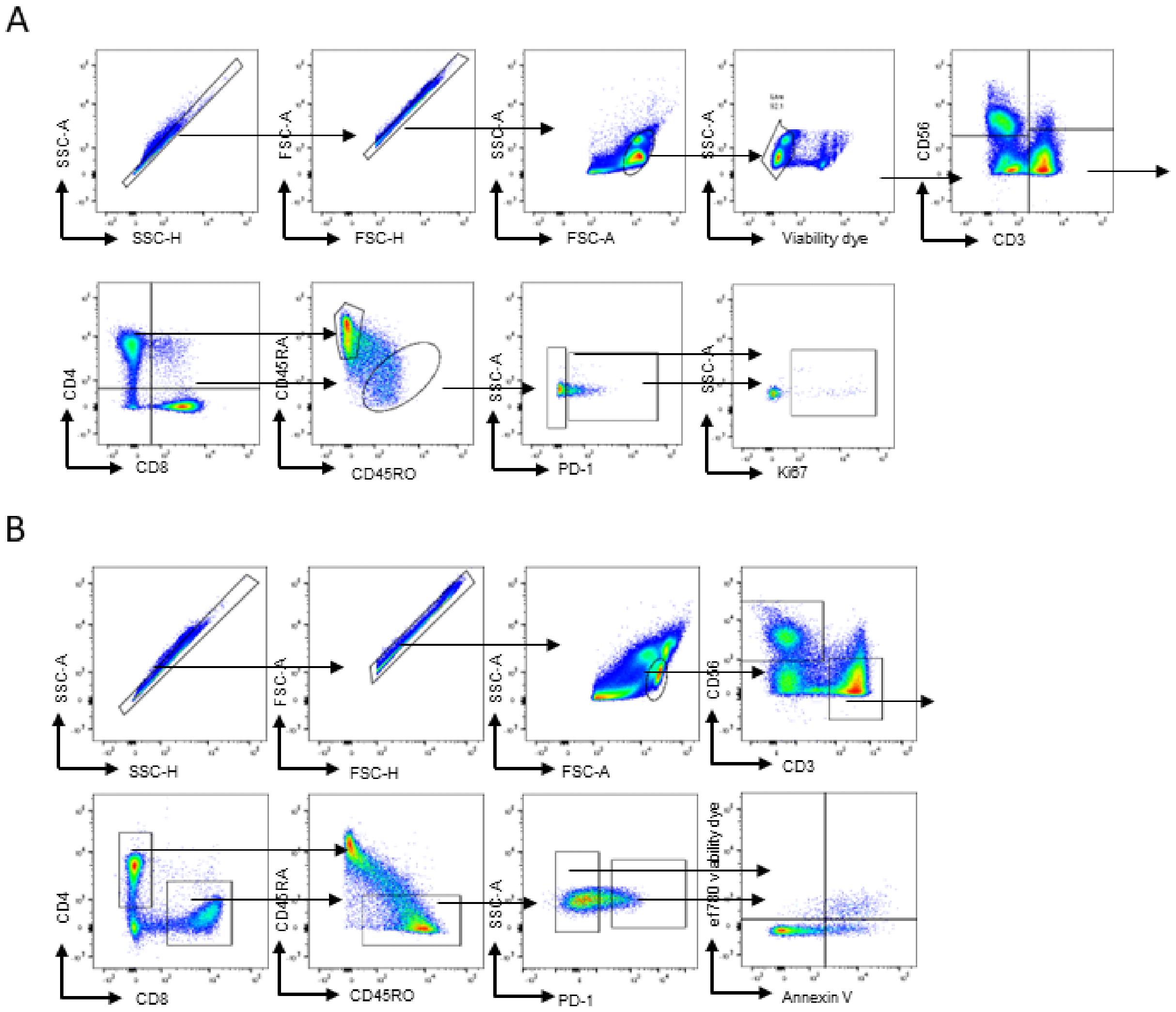

Supplemental Figure 3. A & B) Representative flow cytometry gating for Ki67 and Annexin V staining of hPBMCs.

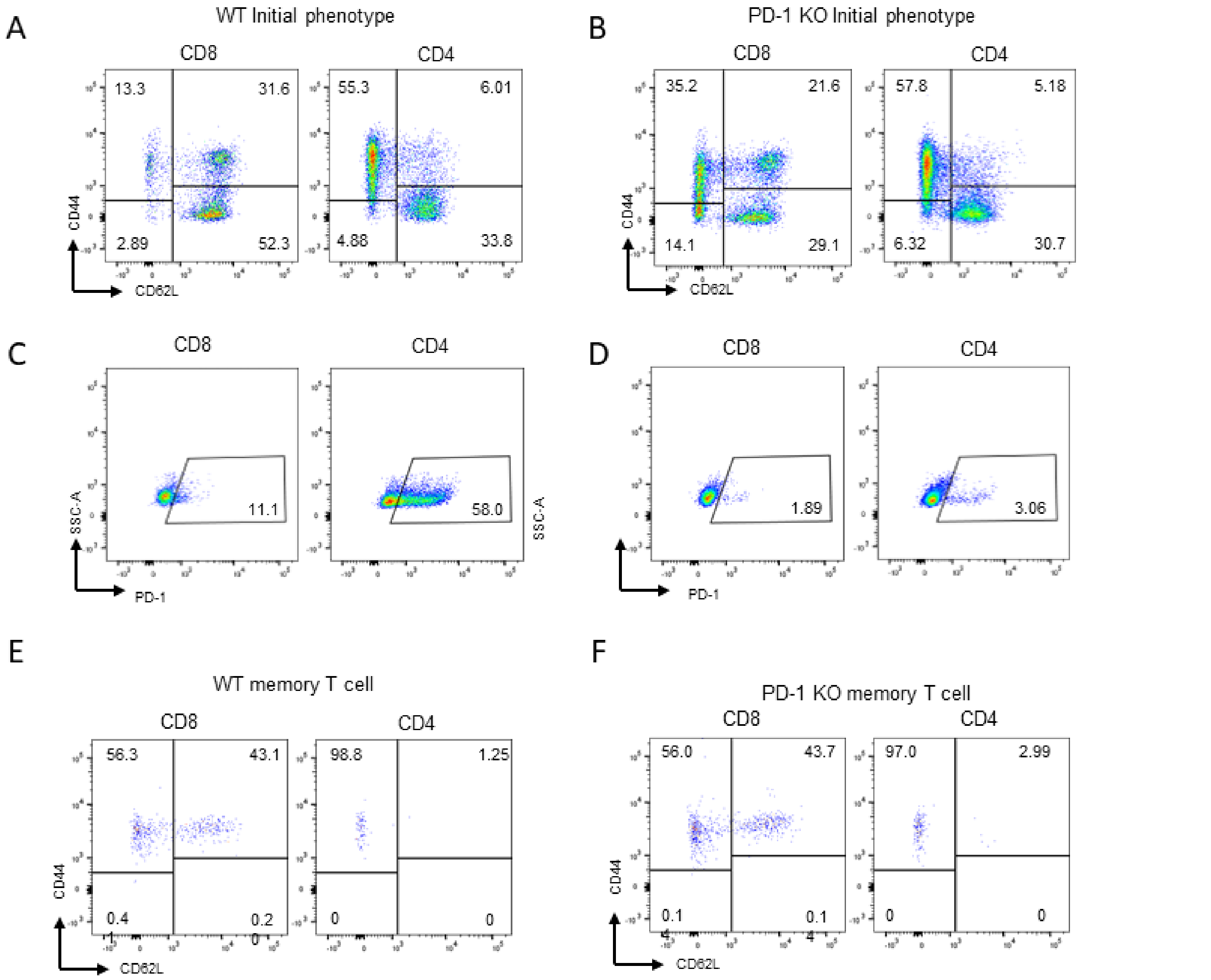

Supplemental Figure 4. A & B) Representative pre-sort memory phenotype of WT and PD-1 KO T cells. C & D) Representative PD-1 staining of pre-sort WT and PD-1 KO T cells. E & F) Representative post-sort memory phenotype of WT and PD-1 KO T cells used for adoptive transfer studies.

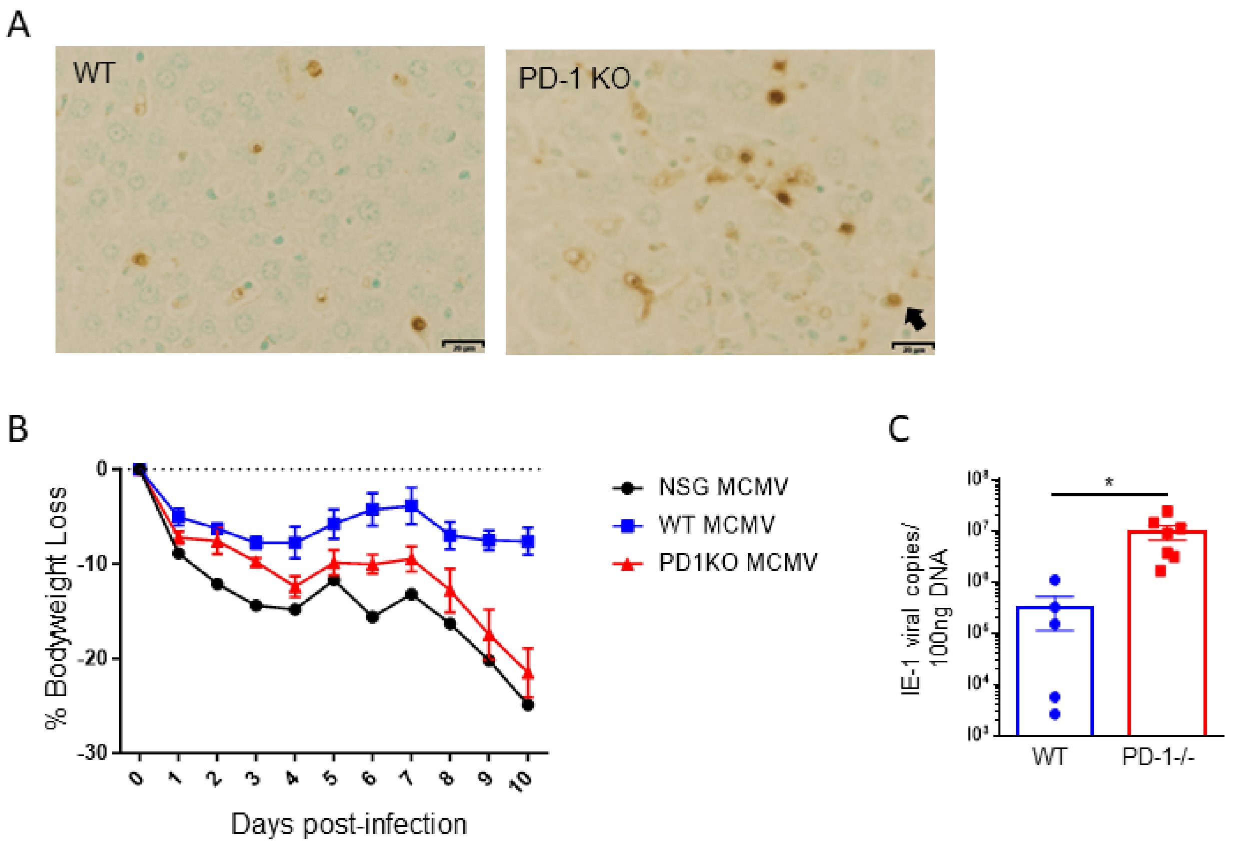

Supplemental Figure 5. A) Representative TUNEL Immunohistochemistry staining of liver tissue from WT and PD-1 KO cell recipients. B) Percent bodyweight loss overtime of NSG without adoptive transfer, WT cell NSG recipients and PD-1 KO cell NSG recipients following infection with MCMV. C) MCMV viral copies in liver at day 11 post MCMV infection in NSG recipients of WT or PD-1 KO cells. Two-tailed unpaired Student's t-test were used to compare two groups (C). *$p<0.05$
